# Effects of Long-Term Temozolomide Treatment on Glioblastoma and Astrocytoma WHO Grade 4 Stem-like Cells

**DOI:** 10.3390/ijms23095238

**Published:** 2022-05-07

**Authors:** Jonas Feldheim, Almuth F. Kessler, Julia J. Feldheim, Ellina Schulz, David Wend, Lazaros Lazaridis, Christoph Kleinschnitz, Martin Glas, Ralf-Ingo Ernestus, Sebastian Brandner, Camelia M. Monoranu, Mario Löhr, Carsten Hagemann

**Affiliations:** 1 Section Experimental Neurosurgery, Department of Neurosurgery, University of Würzburg, D-97080 Würzburg, Germany; jonas.feldheim@uk-essen.de (J.F.); kessler_a1@ukw.de (A.F.K.); julia.feldheim@uk-essen.de (J.J.F.); ellinaschulz@gmail.com (E.S.); david.wend@web.de (D.W.); ernestus_r@ukw.de (R.-I.E.); loehr_m1@ukw.de (M.L.); 2 Division of Clinical Neurooncology, Department of Neurology, University Hospital Essen, D-45131 Essen, Germany; lazaros.lazaridis@uk-essen.de (L.L.); christoph.kleinschnitz@uk-essen.de (C.K.); martin.glas@uk-essen.de (M.G.); 3 Center for Translational Neuro- and Behavioral Sciences, University Hospital Essen, D-45131 Essen, Germany; 4 Department of Neurosurgery, University Hospital Essen, D-45131 Essen, Germany; 5 Division of Neuropathology, Department of Neurodegenerative Disease, UCL Queen Square Institute of Neurology, University College Hospitals NHS Foundation Trust, London WC1N3BG, UK; s.brandner@ucl.ac.uk; 6 Department of Neuropathology, Institute of Pathology, University of Würzburg, D-97080 Würzburg, Germany; camelia-maria.monoranu@mail.uni-wuerzburg.de

**Keywords:** glioblastoma, astrocytoma, IDH, MGMT, therapy, temozolomide, cancer stem cells

## Abstract

Glioblastoma leads to a fatal course within two years in more than two thirds of patients. An essential cornerstone of therapy is chemotherapy with temozolomide (TMZ). The effect of TMZ is counteracted by the cellular repair enzyme O^6^-methylguanine-DNA methyltransferase (MGMT). The *MGMT* promoter methylation, the main regulator of MGMT expression, can change from primary tumor to recurrence, and TMZ may play a significant role in this process. To identify the potential mechanisms involved, three primary stem-like cell lines (one astrocytoma with the mutation of the isocitrate dehydrogenase (IDH), CNS WHO grade 4 (HGA)), and two glioblastoma (IDH-wildtype, CNS WHO grade 4) were treated with TMZ. The *MGMT* promoter methylation, migration, proliferation, and TMZ-response of the tumor cells were examined at different time points. The strong effects of TMZ treatment on the *MGMT* methylated cells were observed. Furthermore, TMZ led to a loss of the *MGMT* promoter hypermethylation and induced migratory rather than proliferative behavior. Cells with the unmethylated *MGMT* promoter showed more aggressive behavior after treatment, while HGA cells reacted heterogenously. Our study provides further evidence to consider the potential adverse effects of TMZ chemotherapy and a rationale for investigating potential relationships between TMZ treatment and change in the *MGMT* promoter methylation during relapse.

## 1. Introduction

Glioblastoma (IDH-wildtype) is amongst the most malignant primary brain tumors in adults [1]. In more than two-thirds of these patients, it leads to a fatal course within two years after diagnosis [2,3]. The effective treatment of these tumors presents a major challenge for multiple reasons: firstly, due to their highly infiltrative growth [4,5] and localization in the brain—one of the most intricate organs—complete resection of glioblastoma is not possible. Secondly, only a limited selection of systemic therapeutical options is available, due to the presence of the blood–brain barrier, which can shield glioblastoma from many of these drugs [6]. The current standard therapy of glioblastoma is still mainly based on a clinical trial published in 2005 that showed a benefit of a triple-therapy consisting of tumor resection, radiotherapy, and chemotherapy with temozolomide (TMZ), compared to resection and radiotherapy alone [7,8]. Such combined surgical and systemic therapy is only transiently effective as glioblastoma tend to recur and develop therapeutic resistance [9].

Cancer stem cells (CSC) are believed to play a major role in tumor initiation as well as recurrence and are thought to be particularly resilient [10,11]. It has been shown in vitro and in vivo that glioblastoma harbor small subpopulations of cells capable of self-renewal and tumor initiation in xenografts [12,13,14]. Therefore, glioblastoma CSCs are an ideal model to study changes in the (epi-)genetics, metabolism and cell behavior of glioblastoma after therapeutic intervention and might be characteristic of recurring tumor cells.

The 2016 update of the World Health Organization (WHO) classification of CNS tumors presented a major paradigm shift that was consolidated in the 5th edition in 2021, [15,16]. While histological features are still an important approach to diagnostic (neuro-) pathology, increasingly molecular features are essential to determine type and grade of tumors, and this has a significant impact on clinical trials and research into glioblastoma [15,17,18,19,20]. It is now established that tumors with a mutation of isocitrate dehydrogenase (IDH) have a molecular profile and thus an origin distinct from IDH-wildtype (IDHwt) glioblastoma. This is underpinned by evidence that tumors that were previously diagnosed as IDH-wildtype astrocytomas corresponding to WHO (2016) grades II or III in fact represent IDHwt glioblastoma and thus are now classified as such, whilst former IDH-mutant glioblastomas have now been renamed into IDH-mutant astrocytomas (CNS WHO grade 4) to reflect a distinct lineage [17,21,22]. These tumors are defined by having morphological high-grade features (necrosis and/or microvascular proliferation) and/or the presence of CDKN2A/B homozygous deletion [16,23]. 

The hypermethylation of the *O^6^-methylguanine-DNA-methyltransferase* (*MGMT*) gene promoter is a molecular marker used in clinical practice. Instead, for diagnostic purposes, the *MGMT* promoter methylation analysis is performed for the purpose of prognostication, i.e., clinical decision-making. The *MGMT* promoter methylation is present in approximately 80% of WHO grade 2 and 35–45% of WHO grade 3 and 4 gliomas [24,25,26]. The *MGMT* gene is located on chromosome 10q26 and encodes a ubiquitously expressed repair enzyme that removes alkyl groups from guanine in the DNA [27]. If the MGMT enzyme is active, it can remove the O-6′ methyl groups introduced by TMZ-chemotherapy, partly repairing the DNA and thereby reducing its therapeutic impact; thus, the downregulation of its expression by the methylation of its promoter is thought to increase TMZ efficiency [27]. Shortly after the initial trial implementing TMZ as a therapeutical standard, Hegi et al. showed a benefit in survival for the subgroup of patients with high *MGMT* promoter methylation [26], a result that has been confirmed subsequently [28,29,30], and the *MGMT* promoter methylation has gained high significance as a prognostic biomarker and can inform therapeutical decisions both at initial diagnosis and at tumor progression [8,31,32,33,34]. In contrast, the recent guidelines of the European Association of Neurooncology [8,35] do not recommend re-evaluating *MGMT* at tumor recurrence. In a comparative literature review and meta-analysis, we showed that the *MGMT* promoter methylation did indeed change significantly in more than 20% of glioblastoma patients [36]. So far, however, the molecular mechanisms behind these changes, as well as their therapeutic implications, remain unclear.

To understand the potential underlying mechanisms, we derived cell lines from one IDH-mutant astrocytoma (CNS WHO grade 4) and two IDH-wildtype glioblastoma and treated them with TMZ to determine the effects of long-term treatment. Our main focus was on (1) the degree of the *MGMT* promoter methylation changes caused by TMZ and their potential implications for further TMZ response; (2) the examination of changes in the tumor cell behavior, such as migration, proliferation, and metabolism, based on the treatment; (3) the evaluation whether and how these changes were dependent of the CSCs’ molecular profile (high or low *MGMT* promoter methylation); and (4) the identification of differences between cells treated by various treatment schemes.

## 2. Results

### 2.1. Long-Term TMZ-Treatment of MGMT+ Cells Had Effects on Cell Proliferation but Did Not Affect Stem-Cell Markers

The experiments using the glioblastoma cell line with unmethylated *MGMT* promoter (0%, MGMT-) and the astrocytoma, IDH-mutant, CNS WHO grade 4 (high grade astrocytoma, HGA) with low levels of *MGMT* promoter methylation (5%) were performed as planned, but the initial treatment period for the glioblastoma cell line with a high degree of *MGMT* promoter methylation (>50%; MGMT+) had to be discontinued. 

Cells in both treatment groups, the 5 days TMZ/23 days recovery scheme (5 d/23 d) and the daily treatment over 6 weeks scheme (6 w), proliferated at very low rates until no cell proliferation was detectable. Once a cell line ceased to proliferate to reach 80% confluency within 14 days, the treatment of the 6 w group was terminated on day 32, and the treatment of the 5 d/23 d group terminated on day 39. The surviving cells from experimental repeats were then pooled to collect sufficient cells for further analysis. In contrast, both the MGMT- and the HGA cells regularly reached confluency, with necrotic cells starting to appear within 3–5 days. No difference was detectable between treatment groups. Proliferation was estimated based on confluency in this phase of the experiment. Therefore, only distinct differences were detectable. 

**Figure 1:**
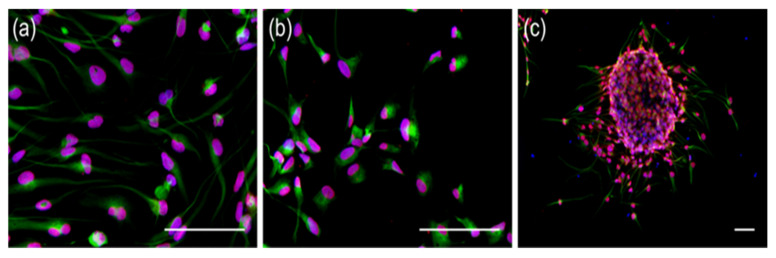
Glioblastoma and HGA stem-like glioma-initiating cells stained positive for SOX-2 and Nestin. Exemplary immunofluorescence staining of SOX-2 (red), Nestin (green), and DAPI (blue) in pre-treatment MGMT+ (**a**), post-5-days/-23 days DMSO treatment HGA (**b**), and monolayer cell cultures and 6 weeks of TMZ treatment MGMT+ neurosphere culture (**c**). The scale bars represent 100 µm each. Abbreviations: HGA, astrocytoma, isocitrate dehydrogenase mutant, CNS WHO grade 4; DAPI, 4′,6-Diamidin-2-phenylindol; MGMT+, glioblastoma cell line with >50% *O^6^-methylguanine-DNA-methyltransferase* promoter methylation; and DMSO, dimethyl sulfoxide.

To establish whether the treatment might influence the CSC characteristics of the cell lines, the immunofluorescence staining of the stem-cell markers sex determining region Y-box 2 (SOX-2) and Nestin was performed on selected samples. Strong co-expression in all specimens before (Figure 1a) and after treatment (Figure 1b,c), regardless of treatment group (5 d/23 d or 6 w, as well as DMSO or TMZ) and cell-line, was detected. Nestin stained cytoplasm, while SOX2 and DAPI colocalised in the nucleus. The expression of stem-cell markers was independent of the original MGMT-status or of the growth pattern in monolayers or tumor spheres. 

### 2.2. TMZ-Induced Proliferation and Migration Was Dependent on the MGMT Promoter Methylation Status

**Figure 2:**
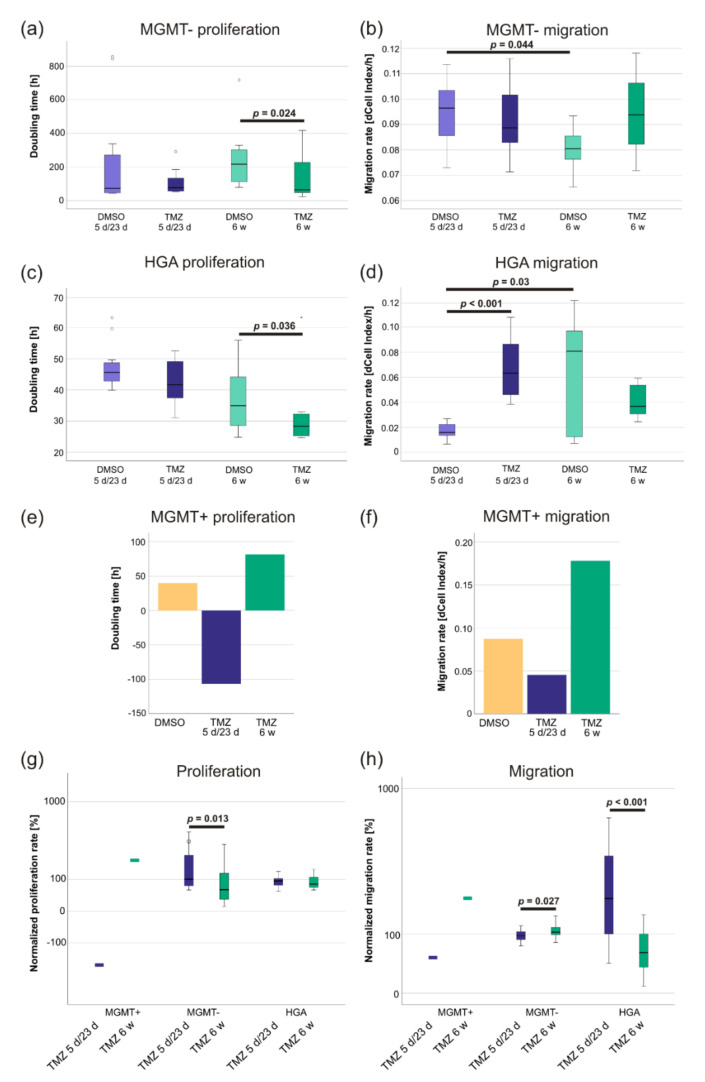
The increased proliferation and migration of TMZ-treated glioblastoma and HGA stem-like glioma-initiating cells was dependent on their *MGMT* promoter methylation status. An xCELLigence device was used to determine the proliferation and migration rate of MGMT- (**a**,**b**), HGA (**c**,**d**) and MGMT+ cell lines. The latter represent pooled samples as their long-term treatment had to be discontinued (**e**,**f**). After normalizing the values to their respective DMSO controls, the cells’ proliferation (**g**) and migration rate (**h**) after the full 8 weeks of therapy and recovery was compared. Abbreviations: HGA, astrocytoma, isocitrate dehydrogenase mutant, CNS WHO grade 4; MGMT, O^6^-methylguanine-DNA-methyltransferase; MGMT-, the glioblastoma cell line without MGMT promoter methylation; MGMT+, the glioblastoma cell line with high MGMT promoter methylation; TMZ, the sample treated with temozolomide; DMSO, the sample treated with dimethyl sulfoxide; 5 d/23 d, the sample of the 5-day-treatment/23-day-recovery-treatment group; and 6 w, the sample of the 6-week-treatment group.

The general migration rate of MGMT- and the doubling time of HGA cells showed a normal distribution (Levene’s test: both *p* > 0.05), while the doubling time of MGMT-(skewness: 2.3, kurtosis: 5.7, Shapiro–Wilk test: *p* < 0.001) and the migration rate of HGA cells (skewness: 0.6, kurtosis: −0.7, Shapiro–Wilk test: *p* = 0.004) did not. In MGMT- cells, 6 w TMZ treatment increased the proliferation rate compared to the corresponding DMSO control (Figure 2a). These cells had a significantly lower doubling time (*p* = 0.024; difference in doubling time: 111.7 h; 95% CI: −17–240 h), while a difference in the 5 d/23 d treatment groups was not observed (Figure 2a). Migration was not affected by TMZ treatment in both treatment groups (both *p* > 0.05) (Figure 2b). However, the MGMT- cells showed a significant difference between the migration rates of both DMSO control groups (5 d/23 d DMSO vs. 6 w DMSO, *p* = 0.044, the difference in the migration rate: 0.015, 95% CI: 0.001–0.029) (Figure 2b).

The proliferation rate of the HGA cells did not significantly change during the 5 d/23 d treatment (TMZ compared to DMSO, *p* > 0.05). In contrast, the doubling time of the cells treated with 6 w TMZ was shorter (mean 11.4 h) than the DMSO control (*p* = 0.036, 95% CI: 0.5–22.0 h) (Figure 2c). However, HGA cells treated with DMSO for 6 w had a significantly higher migration rate (dCell-Index/h) with broad distribution, compared to the cells treated by the 5 d/23 d scheme (*p* = 0.03, the difference in the migration rate: 0.05, 95% CI: 0.009–0.09) (Figure 2d). Interestingly, the treatment with TMZ led to an increase of migration in the 5 d/23 d group (*p* < 0.001, the difference in the migration rate: 0.05, 95% CI: 0.03–0.07) (Figure 2d). In contrast, a decrease of the migration in the cells treated with 6 w TMZ compared to their DMSO control (*p* > 0.05) was observed (Figure 2d) but did not reach statistical significance.

The MGMT+ cells treated with the 5 d/23 d scheme were unable to divide in the small wells of the xCELLigence plates and showed a decreased migration rate (Figure 2e,f). Similarly, cells treated with 6 w TMZ proliferated at less than half the rate (Figure 2e). However, they migrated 2–3 times faster than the DMSO-treated control (Figure 2f).

To directly compare the differences between the 6 w and the 5 d/23 d treatments of the different CSCs, the TMZ-treated specimens were normalized to their respective DMSO controls. The MGMT+ cells of the 6 w TMZ group were able to reproduce on the xCELLigence plate (Figure 2g) and migrated at a higher pace (Figure 2h), while the 5 d/23 d MGMT+ cells did not. Compared to their DMSO controls, 6 w TMZ led to significantly higher proliferation (*p* = 0.013) and migration rates (*p* = 0.027) than 5 d/23 d TMZ treatment in MGMT- cells (Figure 2g,h). Interestingly, the latter appears to be the opposite in HGA cells, where 6 w TMZ led to a significant decrease in the migration rate (*p* < 0.001) (Figure 2g,h).

### 2.3. MGMT Promoter Methylation Changed after TMZ Treatment of HGA and MGMT+ Cells

**Figure 3:**
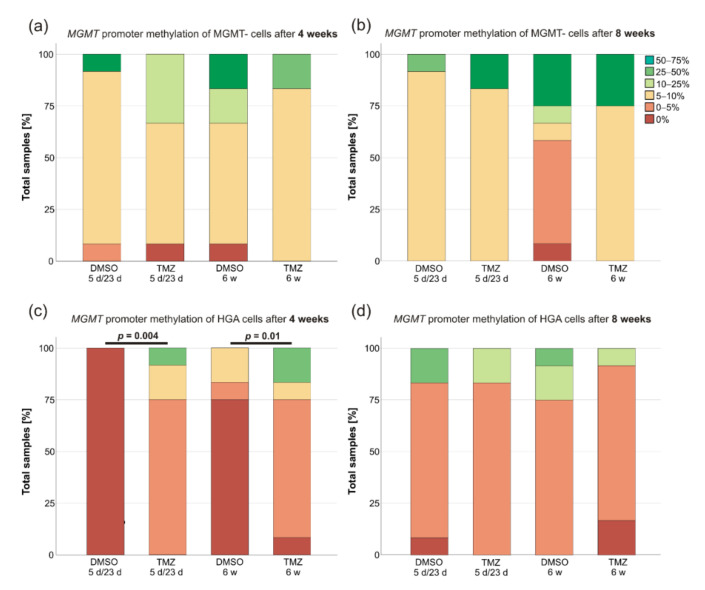
The MGMT promoter methylation changes of the glioblastoma and the HGA stem-like glioma-initiating cells. The MGMT promoter methylation of the MGMT- (**a**,**b**) and HGA (**c**,**d**) cell lines was determined 4 and 8 weeks after 5 d/23 d and 6 w treatment scheme initiation, respectively. As MGMT+ cells treated with TMZ had to be pooled; their results are not shown in this bar-graph. Abbreviations: MGMT, O^6^-methylguanine-DNA-methyltransferase; MGMT-, the glioblastoma cell line without the MGMT promoter methylation; HGA, astrocytoma, isocitrate dehydrogenase mutant, CNS WHO grade 4; TMZ, the sample treated with temozolomide; DMSO, the sample treated with dimethyl sulfoxide; 5 d/23 d, the sample of the 5-day-treatment/23-day-recovery-treatment group; and 6 w, the sample of the 6-week-treatment group.

The *MGMT* promoter methylation status of the CSCs was evaluated 4 weeks after the treatment initiation and again after another 4 weeks. Multiple changes in the degree of the promoter methylation were observed. The only exceptions were MGMT- cells that only slightly increased their degree of the methylation during the course without significant differences between TMZ and DMSO treatment (Figure 3a,b). However, the MGMT promoter methylation did significantly change in either treatment groups of the HGA cell line compared to their DMSO controls after 4 weeks (Figure 3c) (Mann–Whitney-U, 5 d/23 d: *p* = 0.004; 6 w: *p* = 0.01) but not 8 weeks (*p* > 0.05) (Figure 3d). Strikingly, the high degree of the methylation in the MGMT+ cells was maintained in all controls treated with 5 d/23 d or 6 w DMSO (24 out of 24 in the 75–100% range), whereas the cells treated with TMZ had a much lower degree of the MGMT methylation (25–50% in the 5 d/23 d group and 5–10% in the 6 w group).

### 2.4. TMZ-Treatment Increased the Sensitivity of HGA Cells to Renewed TMZ Exposure

**Figure 4:**
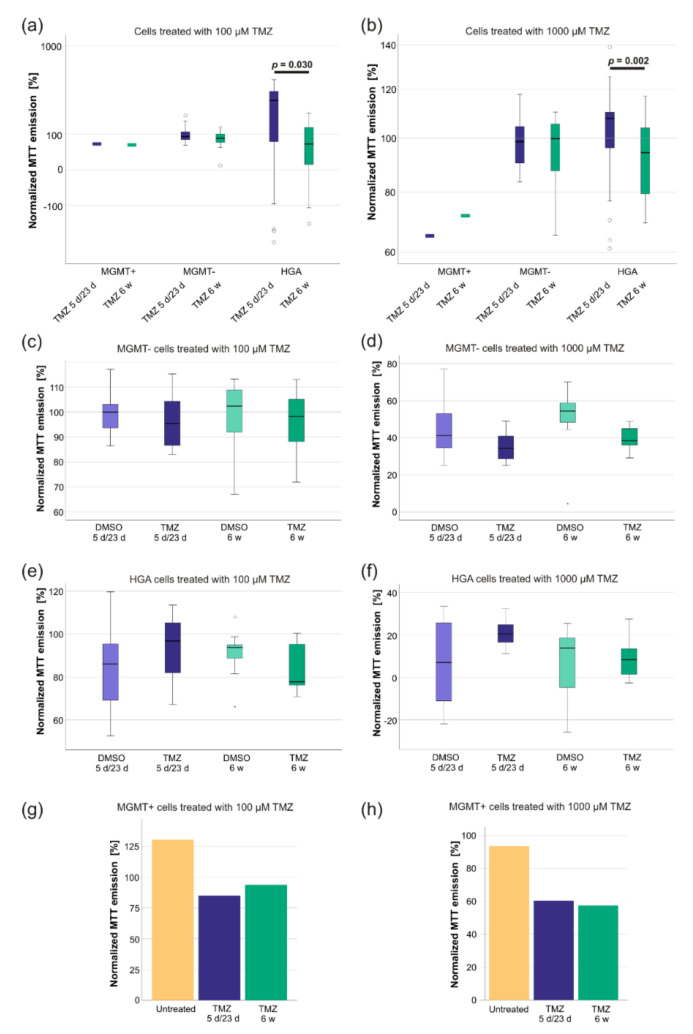
A comparison of the sensitivity of glioblastoma and HGA stem-like glioma initiating cells to renewed short-term TMZ treatment. The cells’ response to renewed TMZ exposure was tested after the total 8-week-long treatment schemes utilizing an MTT-assay by re-exposing the pretreated cells to TMZ concentrations of 100 µM (**a**) and 1000 µM (**b**) for 48 h. The values were normalized to the results of their respective DMSO treatment group. Circles represent outliers. In addition, the metabolic activity of MGMT- (**c**,**d**), HGA (**e**,**f**), and MGMT+ (**g**,**h**) cell lines was determined in comparison to their respective DMSO controls (of the MTT assay). All values were normalized to their positive and negative controls of the MTT-assay. Abbreviations: HGA, astrocytoma, isocitrate dehydrogenase mutant, CNS WHO grade 4; TMZ, the temozolomide treated sample; DMSO, the sample treated with dimethyl sulfoxide; MTT, 5-(3-methyltriazen-1-yl)-imidazole-4-carbox-amide; MGMT+, glioblastoma cell line with >50% *O^6^-methylguanine-DNA-methyltransferase* promoter methylation; MGMT-, glioblastoma cell line without *O^6^-methylguanine-DNA-methyltransferase* promoter methylation; 5 d/23 d, sample of the 5-day-treatment/23-day-recovery-treatment group; 6 w, the sample of the 6-week-treatment group.

Next, we examined if the sensitivity of the cells to renewed short-term TMZ exposure was altered after finalization of the previous treatment schemes. Before treatment, the TMZ response of CSC was in keeping with the prediction according to the MGMT promoter methylation: MGMT- cells had a TMZ-IC50 of 510.3 µM, HGA cells of 910.5 µM, and MGMT+ cells of 35.4 µM. Based on these IC50s, the response on renewed TMZ (100 µM and 1000 µM) exposure was examined utilizing a 5-(3-methyltriazen-1-yl)-imidazole-4-carbox-amide (MTT) assay. Whereas there was no effect on MGMT+ and MGMT- cells, respectively, HGA cells treated with 6 w TMZ responded stronger to an additional short-term TMZ exposure just before the MTT assay (100 µM TMZ: *p* = 0.03; 1000 µM TMZ: *p* = 0.002) (Figure 4a,b).

When comparing the normalized treatment schemes, the MTT emission of the MGMT- cells treated with 100 µM TMZ and 1000 µM TMZ displayed a normal distribution (Levene’s test: *p* > 0.05), as did the MTT emission of the HGA cells treated with 100 µM TMZ (Levene’s test: *p* > 0.05, each Shapiro–Wilk test: *p* > 0.05), but not the MTT emission of HGA cells treated with 1000 µM TMZ (skewness: −0.8, kurtosis: −0.04, Shapiro–Wilk test: *p* = 0.015). However, there were no statistically significant differences in the responses to renewed TMZ exposure between the DMSO and TMZ treatment groups of all cell lines (*p* > 0.05) (Figure 4c–h).

## 3. Discussion

### 3.1. Long-Term TMZ Treatment Promotes a Highly Proliferative Phenotype

The genetic profile of recurrent tumors in most instances shows additional genetic and epigenetic changes, with new passenger, and sometimes also new driver, mutations occurring. These can increase therapy resistance and contribute to the increased risk of recurrence [37]. The two mechanisms may be responsible for such alterations: heterogeneity within the initial tumor, for example, pre-existing therapy-resistant subclones that become dominant due to the selective treatment pressure, and the mutagenic effect of the therapy itself [37]. TMZ has the potential to cause changes in the methylation profile of tumor cells and to introduce new C > T/G > A mutations in critical pathways [38,39,40]. Therefore, newly acquired mutations or the aggressive behavior of the surviving cells might represent the adverse effects of TMZ chemotherapy.

**Figure 5:**
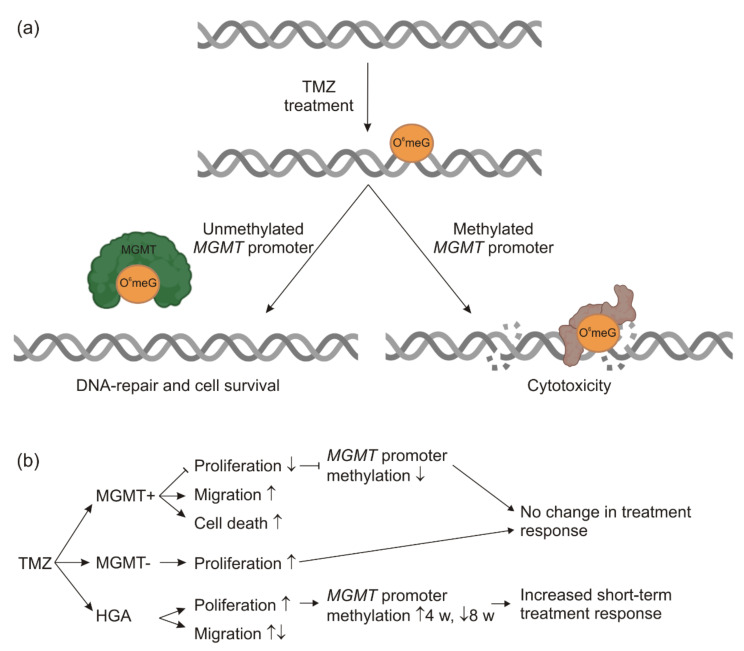
The scheme of TMZ and MGMT effects. In non-cancerous, as well as in glioma cells, TMZ introduces alkylating changes at the DNA, such as O^6^meG methylation. In the case of an unmethylated *MGMT* promoter, the MGMT enzyme is expressed and transfers the methyl-group to itself, repairing the DNA (left). Cells with the methylated *MGMT* promoter do not or only marginally express MGMT. In this case, the DNA damage persists and may trigger apoptosis or cytotoxicity (right). The schematic diagram was partially created with biorender.com (**a**). A summary of the experimental results. ↑ = upregulation, ↓ = downregulation (**b**). Abbreviations: HGA, astrocytoma, isocitrate dehydrogenase mutant, CNS WHO grade 4; MGMT, O^6^-methylguanine-DNA-methyltransferase; MGMT+, the glioblastoma cell line with >50% *MGMT* promoter methylation; MGMT-, the glioblastoma cell line without *MGMT* promoter methylation; O^6^meG, O^6^-methylated Guanine; TMZ, temozolomide; and w, weeks.

In accordance with this hypothesis, MGMT- cells treated with 6 w TMZ significantly increased their proliferation rate, as did the HGA cells with low levels of the *MGMT* promoter methylation. Tumors with low *MGMT* promoter methylation are known to respond poorly to TMZ [26,32], thus raising the question of whether the adverse effects of the treatment might exceed the benefit of the treatment. We did not detect any proliferation of the MGMT+ cells in the 5 d/23 d TMZ group and detected decreased proliferation in the 6 w TMZ group. Still, it remains to be established whether the cells of both groups had already sufficiently recovered from the TMZ treatment. Of note, cells treated with 6 w TMZ showed a proliferation advantage compared to the group with the 5 d/23 d TMZ treatment. This was in keeping with observations for the MGMT- and HGA cell lines, thus suggesting a TMZ-based selection of tumor cells with a higher proliferation rate (Figure 5). The migration rate was inconsistently affected by the TMZ treatment. However, the different migratory behavior might not have led to a survival benefit in our experimental setting. Therefore, it is speculated that highly proliferative cells might have had an advantage due to the selective pressure, whereas high/low migratory cells have not. It is also noteworthy that only in TMZ-treated cells of a low proliferative rate was a high migratory rate observed (Figure 5). This phenomenon has been well described in the literature by the ‘go or grow’ concept, stating that high proliferation may trigger a phenotype with low migration, and vice versa [41].

Surprisingly, the cells treated with TMZ for 48 h at the end of the recovery time did not display any differences in the response measured by the MTT assays. This observation is inconsistent with the published data as it has been well described that TMZ treatment leads to the resistance of glioblastoma primary cells and cell lines [42,43,44]. However, the MTT assay used in this study can over- or underestimate viable samples [45]. Due to the limits of the TMZ solubility, it was not possible to test concentrations higher than 1 mM, although the results indicate that the actual IC50 might have been much higher. 

### 3.2. The TMZ-IC50 of Glioblastoma CSCs Remains to Be Dependent on MGMT Promoter Methylation

It is established that the *MGMT* promoter methylation affects the TMZ-response of glioblastoma [26,28,29,30]. This correlation was confirmed by the strong effects that TMZ treatment had on MGMT+ cells compared to MGMT- and HGA cells. The stem-like cell characteristics of all three cell lines were not altered by the treatment. As exclusively stem-like glioma-initiating cells were selected at the start of the experiments and the proportion of glioblastoma CSC are known to increase in regular glioblastoma tumors during treatment [46], this observation was not surprising.

It is noteworthy that the 5 µM TMZ dose used in the experiments closely resembles the therapeutic concentration measured in the cerebrospinal fluid of the TMZ-treated glioblastoma patients [47]. The IC50 for TMZ of most glioblastoma cell lines, however, is significantly higher. This is particularly true for glioblastoma CSCs that are known for their more resilient behavior and mostly have an IC50 for TMZ of 100 µM and above [46]. Indeed, an IC50 for the MGMT+ cell line was measured that was seven times higher than the clinical cerebrospinal fluid concentration, and the MGMT-s’ and HGAs’ IC50 were more than 100 times higher.

However, their IC50 could only be estimated as no plateau phase was reached in the MTT assay due to the limitations of TMZ solubility. Furthermore, it should be noted that the IC50 is usually measured during a short time window, ignoring the long-term additive effects that might play a significant role in patients treated with TMZ over months [7,32,45,46]. While noteworthy, the discrepancy between the therapeutical dose and the IC50 of the cell lines should, therefore, not be overestimated.

### 3.3. Changes of MGMT Promoter Methylation Were Dependent on the Initial Grade of Methylation and the Duration of TMZ Treatment

It is established that subclones with a preexisting or newly acquired resistance to TMZ within the tumor mass will benefit and prevail when the latter is eradicated with the standard of care [48]. It has also been established that the *MGMT* promoter methylation is a valuable predictor for the TMZ response of both the initial tumor and the relapse [8,31,32,33,34]. Thus, it is surprising that the potential changes of the *MGMT* promoter methylation status as a mechanism to acquire TMZ resistance are rarely acknowledged by guidelines and in neurooncological research [36]. Here, we observed that tumor cells that overcame the effects of TMZ therapy displayed a much lower degree of methylation, which further decreased during treatment. This is in-keeping with clinical data summarized in our recent meta-analysis [36], showing that the *MGMT* promoter methylation can frequently change between the primary tumor and recurrence and that TMZ may play an important (but not the only) role in this. 

As the low *MGMT* promoter methylation already promotes TMZ resistance, it was not surprising that the methylation of MGMT- was not affected by TMZ. Remarkably, the HGA cells treated with TMZ significantly increased in the *MGMT* promoter methylation after four weeks but showed a pattern similar to their control groups after 8 weeks. This interesting phenomenon might be due to the biphasic effect of TMZ on methylation. In the first phase, TMZ can lead to the hypermethylation of the DNA. In the long term, however, the effect is adverse, resulting in global demethylation [49,50].

### 3.4. Conclusions and Outlook

TMZ is a significant part of the standard therapy of almost all glioblastoma and HGA, but TMZ might be of limited use in tumors with the unmethylated *MGMT* promoter [32,51]. Our experiments show that TMZ leads to the unfavorable enrichment of tumor cells with increased proliferation or migration, further challenging the practice of the TMZ therapy of gliomas with the unmethylated *MGMT* promoter (Figure 5). In contrast, TMZ chemotherapy, possibly combined with lomustine, appears to be a useful alternative for glioblastoma with the methylated *MGMT* promoter [52]. In recurrent tumors, the *MGMT* promoter methylation status may aid in the therapeutic decision-making. However, assumptions should not be based on the methylation analysis of the primary tumor alone as the *MGMT* promoter methylation can frequently change [37]. Furthermore, the long-term TMZ treatment has been described to critically interact with the gut microbiome composition, affecting TMZ safety and efficacy, an interaction that might provide valuable new opportunities [53,54,55].

Similarly, TMZ-therapy is standard in the treatment of IDH-mutant astrocytomas [56]. HGA (astrocytomas, IDH mutant, CNS WHO grade 4) can be considered as the same lineage as astrocytomas, IDH-mutant, CNS WHO grades 2 or 3, but they show higher aggressiveness and a higher mutational burden [23]. Some assumptions can be made on their ideal therapy based on subgroup analyses of former clinical trials. However, currently any information on the growth behavior and therapeutic response to TMZ treatment is vital to advise patients before the first randomized controlled clinical trials are conducted. While more insight into TMZ re-exposure and *MGMT* promoter methylation of primary and recurrent HGA CSCs was provided, it is premature to draw a definitive conclusion. It is noteworthy that HGA cells pretreated with TMZ responded more strongly to renewed exposure than their glioblastoma counterparts.

Understanding, avoiding, or even harnessing the effects of TMZ beyond its apparent therapeutic impact, such as new mutations or changes in the methylome, might help one to improve the current standard of care for glioblastoma and HGA patients.

## 4. Materials and Methods

### 4.1. Cell Culture and Cell Biology

The cell lines were selected based on their molecular characteristics: one glioblastoma cell line displayed a high degree of *MGMT* promoter methylation (>50%; MGMT+); one glioblastoma cell line was without *MGMT* promoter methylation (0%, MGMT-); and one cell line represented an astrocytoma, IDH-mutant, CNS WHO grade 4 (high grade astrocytoma, HGA) harboring only low extent of *MGMT* promoter methylation (5%). To acknowledge heterogeneous reactions to treatment, 12 samples of each cell line and treatment group were treated (initially 6, split 1:2 during the course of the experiment). All cell culture experiments were performed under sterile conditions in a laminar flow hood. Depending on the subsequent tests and necessary cell count, the cells were cultured in 75 cm^2^ and 25 cm^2^ culture flasks or 6-well and 12-well culture plates, respectively, (all from Corning Inc., New York, NY, USA) placed in a sterile incubator set at 37 °C, 5% CO_2,_ and 100% humidity (Heracell 240i, Thermo Fisher Scientific Inc., Waltham, MA, USA). For most experiments and standard cultivation, the cells were grown in NeuroCult NS-A Basal medium supplemented with NeuroCult NSA Proliferation Supplement (1:10), 0.2% heparin (1:1000), H Recom EGF (1:5000), Hu Recom bFGF (1:10,000; all from STEMMCELL Technologies Inc., Vancouver, BC, Canada), laminin (1:10,000, Sigma Aldrich/Merck KGaA, St. Louis, MO, USA), and penicillin-streptomycin (1:100, Thermo Fischer Scientific Inc., Waltham, MA, USA). This solution is hence referred to as medium. The cells were split when they reached 80–90% confluency, which was the case approximately every 2–4 days. For this, the media supernatant was collected, the cells were washed with phosphate buffered saline (PBS, Biochrom GmbH, Berlin, Germany) that was collected as well, and then the cells were detached with accutase (STEMMCELL Technologies Inc., Vancouver, BC, Canada) for approximately 4–6 min. The detached cells and collected fluids were centrifuged for 10 min at 200× *g* to remove the accutase. After centrifugation, the supernatant was discarded, the cells were resuspended in 1 mL medium, and the cells were counted with the Countess 2 Automated Cell Counter (Thermo Fischer Scientific Inc., Waltham, MA, USA) according to manufacturer’s instructions. Subsequently, some of the cells were reseeded (300,000/well on 6-well plates, 100,000/well on 12-well plates, 700,000 on 25 cm^2^ culture flasks, and 2,100,000 on 75 cm^2^ culture flasks).

### 4.2. Laminin Coating

Multiple experiments required the pretreatment of surfaces (well plates, experiment vessels, etc.) with laminin for the cells to attach properly. Therefore, laminin from the Engel–breth–Holm–Swarm murine sarcoma basement membrane (Sigma Aldrich/Merck KGaA, St. Louis, MO, USA) was diluted in PBS. The surfaces were covered with this solution, incubated for at least 2 h at 37 °C, and washed twice with PBS for either direct usage or storage overnight at 4 °C for further use. Surfaces treated this way are referred to as ‘prelaminated’ in the following text. To ensure sufficient cell attachment in the migration assays, the gold electrodes were laminated on the flat side of the top part of the CIM plate of the xCELLigence RTCA DP device (ACEA Biosciences Inc./Agilent, Santa Clara, CA, USA). The top part of the CIM plate was placed upside down in a sterile box, and laminin drops of 50 µL held by the water tension were pipetted on top of each of the 16 gold electrodes. After 2 h of incubation at 37 °C/5% CO_2_, they were washed twice with PBS using a similar procedure.

### 4.3. Double-Fluorescence Staining

To stain the cells and spheres, respectively, 10,000 cells of each cell line were seeded on untreated (spheres) or prelaminated (cell monolayer) coverslips and incubated overnight to allow the cells to adhere properly. Being seeded on an unlaminated surface caused cells to form spheres instead of growing as a monolayer. Otherwise, spheres and monolayer-cells were treated equally and are both referred to as ‘cells’ in the following section. On the following day, the cells were washed cautiously with Tween20 wash buffer (Tween20 from Carl Roth GmbH + Co. KG, Karlsruhe, Germany, dissolved 1:1000 in PBS). Subsequently, the cells were fixed by incubating them with paraformaldehyde (PFA, Merck KGaA, St. Louis, MO, USA) for 10 min at room temperature and washing them three times with ice-cold PBS. To make the cells permeable for the staining antibodies, they were incubated with TritonX-100 (Sigma-Aldrich/Merck KGaA, St. Louis, MO, USA) for 10 min at room temperature before being washed three times for 5 min with the Tween20 wash buffer. Unspecific binding sites were blocked with a blocking solution. Then, the primary antibodies ab137385 (anti-Sox2, rabbit, diluted 1:50, Abcam plc, Cambridge, UK) and MAB5326 (anti-Nestin, mouse, diluted 1:200, Merck Millipore/Merck KGaA, St. Louis, MO, USA), diluted in the blocking solution, were applied and incubated overnight at 4 °C.

The next day, the slides were washed three times for 5 min in Tween20 wash buffer before the secondary antibodies were applied. The specimens were protected from light for all further steps. The Goat anti-Rabbit IgG (H+L), the Highly Cross-Adsorbed Secondary Antibody, the Alexa Fluor Plus 555 and the Goat anti-Mouse IgG (H+L), the Highly Cross-Adsorbed Secondary Antibody, and the Alexa Fluor Plus 488 (both diluted 1:400 in PBS with 1% BSA, both from Thermo Fisher Scientific Inc., Waltham, MA, USA) were used and incubated for one hour at room temperature. Afterwards, once again the cells were washed three times for 5 min in Tween20 washing buffer before the glioblastoma and HGA stem-like glioma-inducing cells were embedded with Fluoroshield mounting medium with DAPI (Abcam plc, Cambridge, UK) and stored at 4 °C. Finally, the specimens were photographed and processed as previously described [57], with a LEICA DMI 3000 B microscope and LAS V4.5 software (camera objective: 10× or 40×; exposure: 25 ms; gain: 1.0×; gamma: 1; otherwise standard settings; both from Leica Microsystems GmbH, Wetzlar, Germany).

### 4.4. Treatment

**Figure 6:**
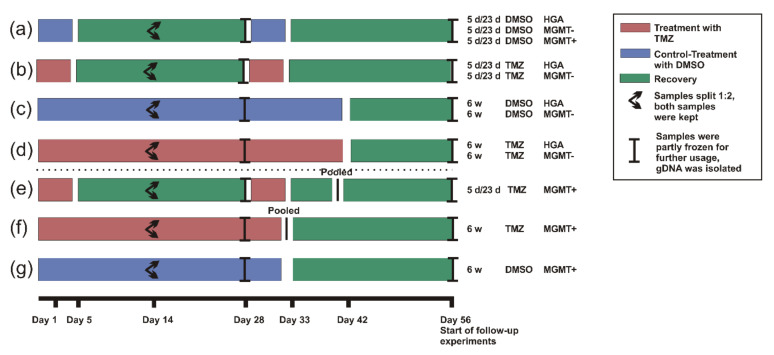
The scheme of the experimental setup to determine the behavioral and molecular changes in the glioblastoma and HGA stem-like glioma-initiating cells with different *MGMT* promoter methylation status. Three glioblastoma stem-like glioma-initiating cell lines (MGMT+, MGMT-, and HGA) were treated with TMZ or DMSO as a control for 8 weeks in two different schemes reflecting the standard of care. Cells were either treated with 2 cycles of DMSO (**a**) or TMZ (**b**) given for 5 days followed by 23 days of recovery (5 d/23 d). A second set of cells was treated continuously with DMSO (**c**) or TMZ (**d**) for 6 weeks followed by a 2-week recovery (6 w). Due to the strong effects of TMZ, the treatment of the MGMT+ cells had to be discontinued after 39 days (5 d/23 d scheme, (**e**)) and 32 days (6 w scheme, (**f**)), respectively. The surviving cells of the experimental repetitions were pooled to secure their survival (**e**,**f**). The treatment of the DMSO groups was stopped accordingly; however, individual samples were kept (**a**,**g**). The cells’ changes in proliferation, migration, therapeutic response, and molecular characteristics (*MGMT* promoter methylation status) caused by the TMZ chemotherapy were determined in follow-up experiments. Abbreviations: 5 d/23 d, 5 days/23 days treatment scheme; 6 w, 6 weeks treatment scheme; HGA, astrocytoma, isocitrate dehydrogenase mutant, CNS WHO grade 4; MGMT+, glioblastoma cell line with >50% *O^6^-methylguanine-DNA-methyltransferase* promoter methylation; MGMT-, glioblastoma cell line without *O^6^-methylguanine-DNA-methyltransferase* promoter methylation; TMZ, temozolomide; DMSO, dimethyl sulfoxide; and gDNA, genomic desoxyribonucleic acid.

To assess the effects of long-term TMZ treatment on the cells, two different treatment settings were chosen with one DMSO control group each (Figure 6). One group was treated daily with 5 µM TMZ (Sigma-Aldrich, Merck KGaA. St. Louis, MO, USA) at approximately the same time for 6 weeks (as applied to most glioblastoma patients directly after the initial surgery), followed by a recovery period of 2 weeks in which the cells were cultured in medium as described above. The second group was treated with 5 µM TMZ for two cycles consisting of 5 days of treatment and 23 days of recovery under normal conditions (as applied to patients after concomitant radiochemotherapy). Controls were incubated with 0.01% of the solvent reagent DMSO (Carl Roth GmbH + Co KG, Karlsruhe, Germany) in the same schemes (Figure 6). Most of the analyses were performed after the whole period of 8 weeks, except for the determination of the MGMT promoter methylation that was done at two different time points (4 and 8 weeks).

The cells were carefully monitored during the treatment period and not split before the first sight of necrotic cells, which was approximately every 3–5 days. The remaining cells were discarded, except after 15 days when we kept a second sample of all specimens. Due to the treatment effects on the MGMT+ cells, the scheme had to be adapted slightly. The treatment was discontinued, and all 12 samples of the respective experimental TMZ groups were pooled, to allow the cells that were closest to TMZ resistance to recover in normal medium. Altogether, the MGMT+ cells were cultured for a total of 8 weeks. In the DMSO group, only the DMSO treatment was discontinued and the individual samples were kept (Figure 6). The genomic DNA (gDNA) was isolated from all samples to determine the *MGMT* promoter methylation as described elsewhere [36]. However, in contrast to the other cell lines, the migration, proliferation, and therapeutic response of the treated MGMT+ cells were compared to the initial untreated cell line instead of TMZ vs. DMSO. As the MGMT+ cells treated with TMZ had to be pooled, the observations would otherwise have been biased by the inhomogeneous sample numbers.

### 4.5. Isolation of gDNA

The gDNA was isolated with the Nucleo Spin DNA purification kit (Macherey Nagel GmbH & Co. KG, Düren, Germany), according to the manufacturer’s instructions. All solutions except the ethanol (Carl Roth GmbH + Co KG, Karlsruhe, Germany) were used as sold with the kit. Briefly explained, up to 1x10^7^ cells were harvested and counted as described above, washed once with PBS, and resuspended in 200 µL T1 buffer. Then, 25 µL Proteinase K and 200 µL buffer B3 were added, and the suspension was mixed and incubated at 70 °C for 10 min. Afterwards, 210 µL ethanol (100%) was added, and the mixture was pipetted onto the column and centrifuged for 1 min at 11,000× *g*. The flowthrough was discarded; 500 µL buffer BW added; the specimens were centrifuged for 1 min at 11,000× *g*; the flowthrough was discarded; 600 µL of buffer B5 was added; and the specimens were centrifuged again for 1 min at 11,000× *g*.

Finally, the flowthrough was discarded, the column was centrifuged for 1 min at 11,000× *g* to let the membrane dry, and the DNA was dissolved in 100 µL buffer BE (1 min incubation at room temperature) before the column was placed into a sterile 1.5 mL tube and centrifuged for 1 min at 11,000× *g*. The concentration was measured and the quality of the DNA evaluated with a Biophotometer D30 and µCuvette G1.0 (both from Eppendorf AG, Hamburg, Germany). The DNA was stored at −20 °C until further processing was carried out.

### 4.6. Detection of MGMT Promoter Methylation by High-Resolution Melting PCR (HRM)

The MGMT promoter methylation was determined by utilizing high-resolution melting PCR (HRM) as previously described [58]. Briefly explained, bisulfite conversion was performed on the extracted gDNA and human controls with the Bisulfite Conversion Kit. Then, the samples were amplified by PCR, and subsequently their melting curve was determined. Each reaction mixture contained 10 µL Melt Doctor™ HRM Master Mix, a 20 ng DNA template, and 5 pM of each primer (forward: 5′-GCGTTTCGGATATGTTGGGATAGT-3, reverse: 5′-CCTACAAAACCACTCGAAACTACCA-3′) and was filled up with RNAse-free water to a total volume of 20 µL. Then, the DNA was amplified with the StepOnePlus PCR Cycler under the following conditions: activation at 95 °C for 10 min, and 45 cycles of amplification consisting of 15 s at 95 °C and 1 min at 60 °C. The final elongation took part at 95 °C for 10 s and 60 °C at 1 min, followed by 15 s at 95 °C and 15 s at 60 °C (all materials and devices were applied from Thermo Fisher Scientific., Waltham, MA, USA).

### 4.7. Cell Viability

The half-maximal inhibitory concentration (IC50) was assessed for TMZ by performing MTT assays (cell-proliferation kit I, Hoffmann–La Roche AG, Basel, Switzerland) to measure the cells’ viability and metabolism, and 10,000 HGA and MGMT- or 5000 MGMT+ cells were cultured under standard conditions in a 96-well plate. After 72 h, the medium was removed and the cells were treated with TMZ (diluted in medium with 1% DMSO), 10% DMSO (as a control for maximum treatment effect), and medium containing 1% DMSO (as a control for no TMZ effect). The DMSO was adjusted in all solutions except the positive control to a level of 1%, to avoid any bias caused by the solvent. The 1% DMSO did not alter the cells’ viability compared to sole medium. All three cell lines were treated with a graded series of TMZ (5 µM-1000 µM) to determine the IC50.

Originating from the initial results, two different TMZ concentrations were chosen to test on the cells that had completed the two treatment schemes, including the final recovery period (Figure 6): 100 µM TMZ (the concentration with a traceable effect, but not a maximum inhibition on all cells, which was therefore suitable to compare the treatment effect on all three cell lines) and 1000 µM TMZ (the maximum concentration, due to a limit of solubility, to assess the maximum treatment effect), as well as 10% DMSO and 1% DMSO as controls. After 48 h of treatment, the 10 µL MTT labeling reagent and 4 h later 100 µL solubilization buffer (from the cell proliferation kit I) were added. Then, the plates were stored at 37 °C/5% CO_2_ and measured the next day in a microplate reader Tecan sunrise (Tecan Group AG, Männedorf, Switzerland) according to the manufacturer’s instructions.

### 4.8. Proliferation and Migration

The proliferation rate and migratory behavior of the glioblastoma and HGA cells were determined using the real-time xCELLigence RCTA DP System that was placed into an incubator (37 °C, 5% CO_2_, 100% humidity). In preliminary tests, the optimal cell count, the condition, and the time frame were determined for each of the cell lines and experiments and consequently applied as outlined below. The pretreated cells were thawed and cultured under standard conditions for seven days to allow them to recover before the start of the experiments.

On the seventh day, to test for proliferation, the wells of a prelaminated E-Plate 16 PET were filled with 100 µL of medium each, and the plates were incubated for at least 1 h in the xCELLigence device, before the background was measured. Meanwhile, the cells were harvested and counted as described above. Afterwards, 10,000 MGMT+ cells, 20,000 MGMT- cells, or 40,000 HGA cells were seeded in 100 µL medium on prelaminated E-Plates and incubated for 30 min at room temperature to allow the cells to attach to the plate evenly.

Afterwards, the E-Plates were placed back into the xCELLigence device, and the measurement was started for 100 h with intervals of 15 min.

To measure cell migration, CIM-plates 16 were used; 160 µL medium with supplements were filled inside the wells of the lower part of the CIM plate, and then the upper part was placed onto the lower part, carefully ensuring that nothing interrupted the contact of the prelaminated gold electrodes to the medium (e.g., air bubbles). Subsequently, the upper side of the electrode was laminated as described above, the wells were filled with 50 µL of supplement-free NeuroCult NSA-Basal medium, and the plate was incubated in the xCELLigence device. After 1 h, the background equilibration was started, the glioblastoma and HGA cells were seeded onto the CIM plate (80,000 cells of all cell lines), and the measurement was started for 20 h.

### 4.9. Bioinformatics Analyses

The HRM melting curves were analyzed with the High-Resolution Melting Software (Thermo Fisher Scientific Inc., Waltham, MA, USA), by comparing the samples with the controls by calculating the “line of best fit”. The samples were distributed in six categories (0%; 0–5%; 5–10%; 10–25%; 25–50%; 50–75%; and 75–100%) with a finer distribution in the lowest quartile, as is common in established clinical tests [36].

**Figure 7:**
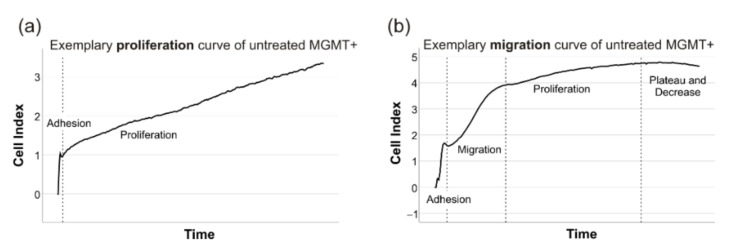
The exemplary xCELLigence curves to determine the proliferation and migration. The cells’ proliferation was measured by determining the mean doubling time after the adhesion period and while the cell index displayed the characteristic constant linear slope (**a**). Similarly, the cells’ migration rate was determined in CIM-plates by measuring the cell index’s slope in a timeframe after the adhesion period had taken place and before cells reached a linear constant slope resembling proliferation (**b**).

The xCELLigence files were analyzed with the RTCA Software 1.0 (ACEA Biosciences Inc./Agilent, Santa Clara, CA, USA). The cells’ proliferation was measured by determining the mean doubling time of two technical replicates (repeated in triplicates if not congruent) in an artifact-free time window of 40–50 h, where cells’ adherence was completed and before the cell index deviated from a linear growth and reached a plateau (Figure 7a). Due to different proliferation rates, these time windows had to be adapted individually. However, the chosen timeframe was mainly dependent on the examined cell line (MGMT+ and HGA: mostly 10–50 h, MGMT-: mostly 30–80 h). Similarly, the cells’ migration rate was determined in CIM-plates by measuring the cell index’s slope in a timeframe, where cells’ adherence was completed and before the linear increase due to cells’ proliferation took part (mostly 1–10 h in all cell lines) (Figure 7b).

To measure the response to TMZ, the optical emission of the MTT samples treated with TMZ was assessed, normalized to the positive and negative control, and logarithmically transformed to determine the half-maximal inhibitory concentration (IC50).

### 4.10. Statistical Analyses

The statistical comparisons and tests were performed with IBM SPSS Statistics 25 (IBM Corporation, New York, NY, USA). The normal distribution was examined by skewness, kurtosis, the Kolmogorov–Smirnov, and the Shapiro–Wilk test. Normally distributed values were further examined by *t*-test (two groups), ANOVA (more than two groups) with Scheffe’s procedure, or Dunnet-T3 as posthoc test depending on Leven’s test to assess the equality of variances and Pearson’s Rho (correlation). The non-normally distributed variables were further compared by Wilcoxon’s (two groups), the Kruskal–Wallis test (more than two groups) with Dunn–Bonferroni as a post-hoc test, and Spearman’s Rho (correlation) as described elsewhere [57,58]. To determine the differences between the effect of 5 d/23 d and 6 w treatment with TMZ, all values were normalized to their respective DMSO control (set as 1.0). 

## Data Availability

All data are contained within the manuscript. Raw data are available on reasonable request from the corresponding author.
